# Supplementary material for: An Enzyme-Linked Immunosorbent Assay for the Detection of Mitochondrial DNA–Protein Cross-Links from Mammalian Cells
Source: DNA (Basel). Author manuscript; Available in PMC 2023 Aug 18. (PMC10438828; doi:10.3390/dna2040019)
Supplement: Supplementary material [file NIHMS1863604-supplement-Supplementary_material.pdf]

DNA

Article

An Enzyme-Linked Immunosorbent Assay for the Detection of Mitochondrial DNA–Protein Cross-Links from Mammalian Cells

Wenyan Xu[1] and Linlin Zhao[1,2,*]

[1] Department of Chemistry, University of California Riverside, Riverside, CA 92521, USA
[2] Environmental Toxicology Graduate Program, University of California Riverside, Riverside, CA 92521, USA
* Correspondence: Tel: +1-951-827-9081; linlin.zhao@ucr.edu

Supplementary Materials

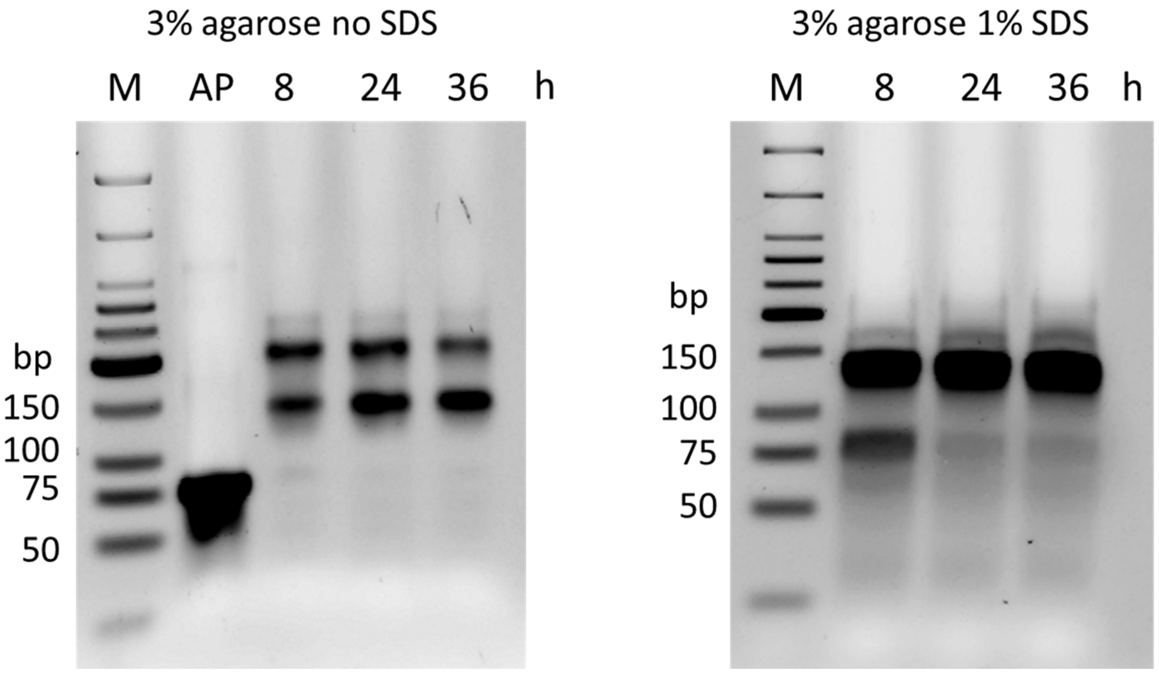

Figure S1. Cross-linking reactions between TFAM and 80bp AP-DNA monitored by agarose gel. Left panel is an agarose gel electrophoresis performed without SDS. The right panel is an agarose gel electrophoresis performed in the presence of 1% SDS. M, DNA molecular weight ladder (New England Biolabs, cat No. B7025); AP, 80bp AP-DNA substrate; reaction times, 8, 24, 36 hours.

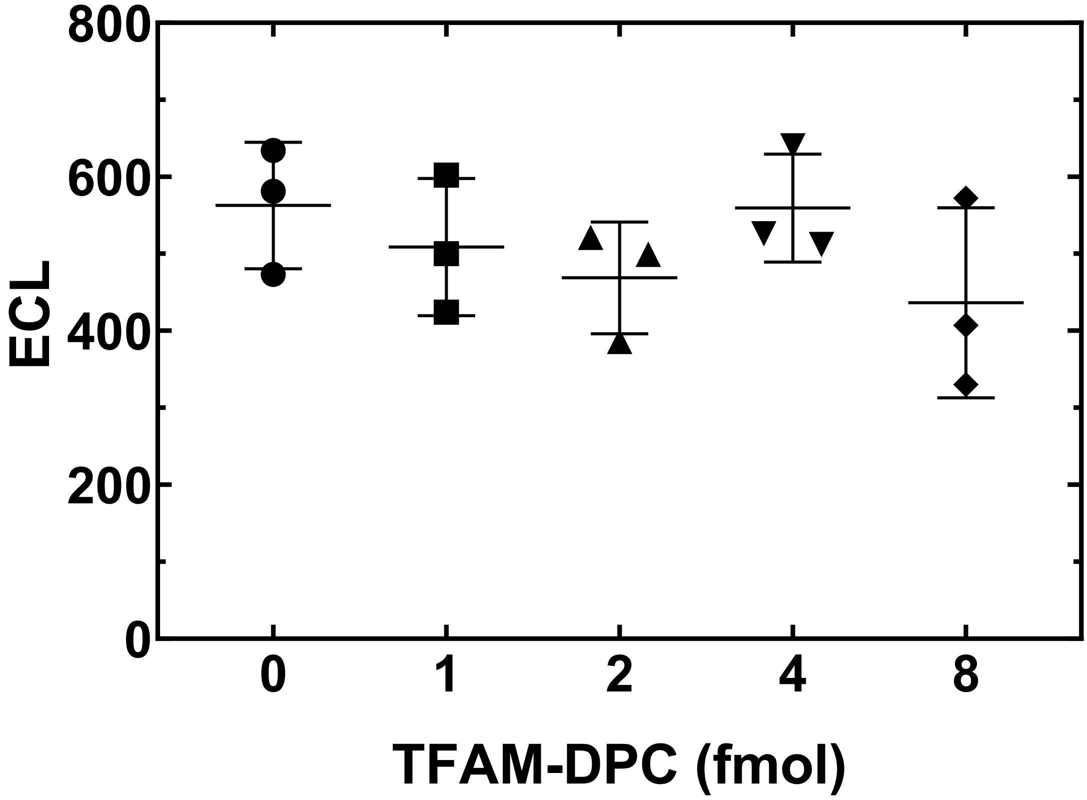

Figure S2. The enhanced chemiluminescent signals obtained from a microplate with an untreated polystyrene surface when different amount of TFAM-DPCs were coated onto the plate. .

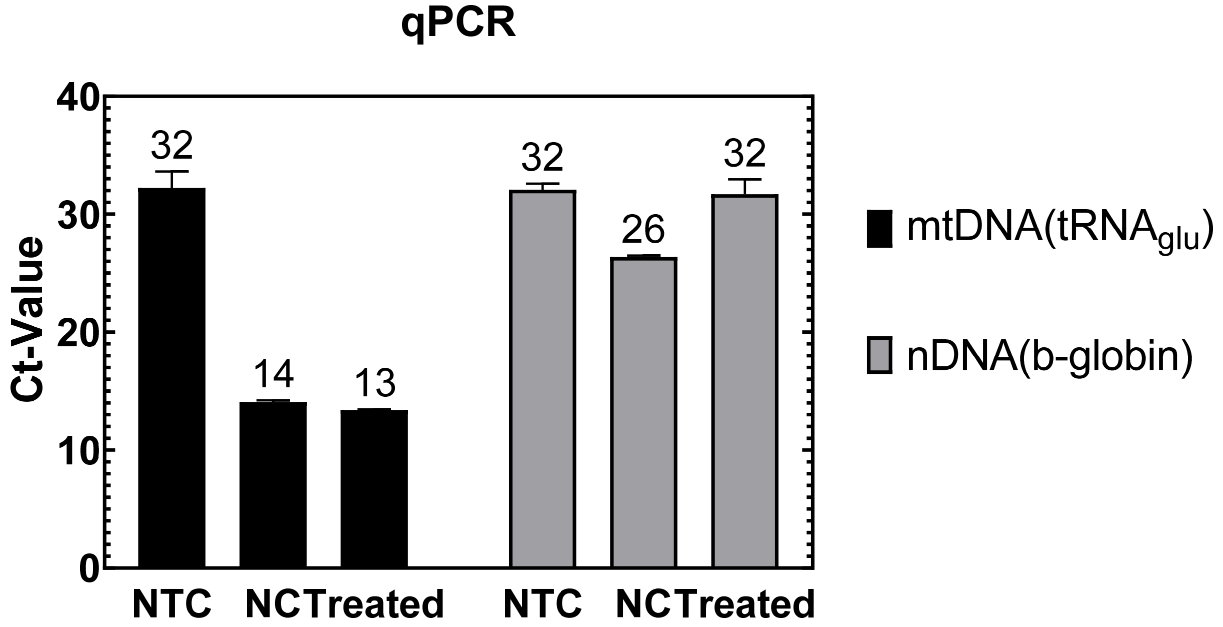

Figure S3. Ct-values from turbonuclease treated mitochondria (Treated) or untreated mitochondria (NC) indicating the removal of nuclear DNA while mitochondrial DNA remained intact. NTC, non-template control.

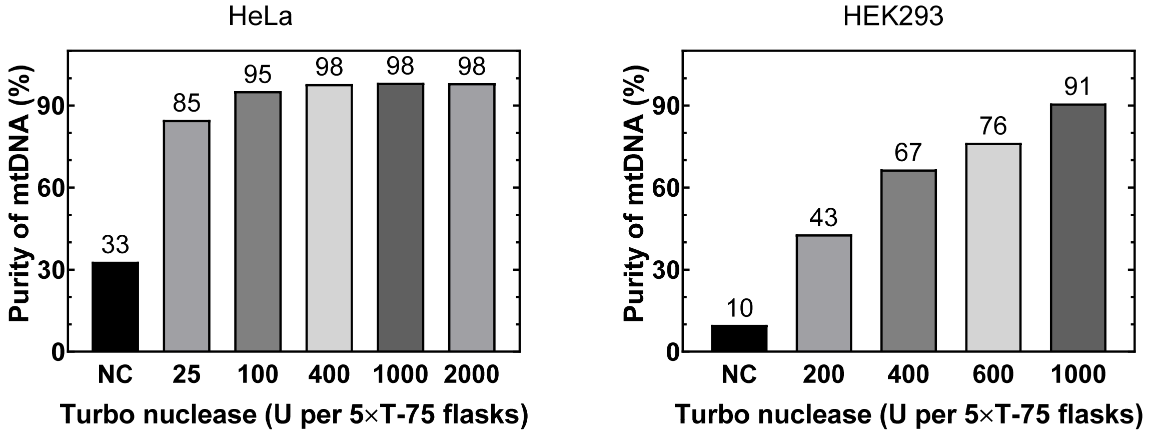

Figure S4. Evaluation of mtDNA purity (based on qPCR data) under different amounts of turbonuclease with mitochondria from HeLa cells and HEK293 cells.
